# Models of Cognition and Their Applications in Behavioral Economics: A Conceptual Framework for Nudging Derived From Behavior Analysis and Relational Frame Theory

**DOI:** 10.3389/fpsyg.2019.02418

**Published:** 2019-11-01

**Authors:** Marco Tagliabue, Valeria Squatrito, Giovambattista Presti

**Affiliations:** ^1^Cultural Selection and Behavioral Economics Lab, Department of Behavioural Sciences, Faculty of Health Sciences, OsloMet – Oslo Metropolitan University, Oslo, Norway; ^2^Kore University Behavioral Lab, Faculty of Human and Social Sciences, Kore University, Enna, Italy

**Keywords:** choice behavior, cognition, contingencies of reinforcement, decision making, IRAP, nudging, relational frame theory

## Abstract

This paper puts forward a rounder conceptual model for interpreting short- and long-term effects of choice behavior. As a further development of dual-process theory, Kahneman (2003) distinguished between intuition and reasoning, which served as the respective precursors of the cognitive processing systems 1 and 2. We maintain that they reflect the more rigorous distinction between brief and immediate and extended and elaborated relational responding, which may be reinterpreted through an analysis of their functional properties. Repertoires of relational responding are offered by the multi-dimensional multi-level model. Specifically, we provide a conceptual account of how nudging, or the manipulation of environmental contingencies, works on the creation and modification of relational framing. Educative nudges, or boosts, are a subset of nudges that may more easily maintain target choice behavior in the future. The central role of verbal behavior is essential toward formulating rules, which inform and guide choice behavior over time. Although nudges are traditionally regarded as System 1-steered aspects, they are herein regarded as cues for responding to relational frames, which may induce System 2-steered aspects. We suggest adopting the implicit relational assessment procedure (IRAP) to inform how coherent and immediate responding to novel relational responding may occur in the presence of choice behavior. Several examples are included to support the claim of encompassing relational responding and choice behavior. We address the instances of consumer behavior, stereotypy and prejudices, eating behavior, and overcoming cognitive biases. The conclusions depict a promising way forward for the study of choice: an improved model for interpreting and overcoming human errors, due to changes in the contingencies of behavior.

## Introduction: The Need for an Enhanced Conceptual Model

As nudge-focused research receives unprecedented attention, we question the comprehensiveness of the conceptual models that sustain the applications of nudging interventions. Nudging refers to the practice of manipulating the environmental and social contingencies of choice behavior, without delivering punishments and rewards. Nevertheless, some scholars do not seem to sustain the universality of nudges (e.g., Marchiori et al., 2017), their ethical justification among users (e.g., Sugden, 2017), and their favorable reception across national populations (e.g., Reisch and Sunstein, 2016; Sunstein et al., 2017). This may denote a property of context dependency that is embedded in nudging interventions.

Behavioral analysis (BA) has a long-standing conceptual and empirical tradition of studying and explaining behavior acquisition and modification. We submit that BA and its contemporary extension of contextual behavioral science (CBS) may be able to provide an overarching conceptual model for nudging that represents more than a simple tactic or a batch of incoherent techniques aimed at overcoming behavioral and cognitive biases (Hansen, 2017). We focus on the property of *context interdependency*, as a common feature to both nudge theory (Thaler and Sunstein, 2003, 2008) and relational frame theory (RFT; Hughes and Barnes-Holmes, 2016). As we use the term, context interdependency refers to the property of an operant [i.e., a form of learned (choice) behavior] to be functionally interrelated with (i.e., influencing and being influenced by) its environmental contingencies, which in several instances are *social*. These reciprocal environmental interactions are particularly useful to providing a broader reference model and interpreting why nudges work, but not necessarily all the time.

In behavioral economics (BE), there is no conceptual model that connects cognition and its proposed applications. BE is an alternative to neoclassic economic theory, insofar as it assumes that agents make good enough choices, rather than best choices. Prospect theory (Kahneman and Tversky, 1979), construal level theory (Trope and Liberman, 2010), and the framework of affective forecasting (Hoerger et al., 2010) are descriptively relevant and valuable models within BE. For example, the corresponding decisional processes of these models, loss aversion, abstracting distal objects, and affective prediction are able to account for deviations from normative and pre-established values (i.e., utility). However, they do not account for deviations from selective and contextual environmental contingencies.

Thus, changes in behavior in the short and long terms do not find exhaustive explanations. BA and RFT traditions show how these changes occur and may give the connection between cognitive processes and their applications in the form of interventions on nudging choice behavior and decision making in the broader contextual vision of CBS (Hayes et al., 2012).

The aim of this article is not to add any new elements to the current framework. Rather, we propose a perspective that integrates human cognition and its applications into an encompassing behavioral model of decision making. This vision integrates epistemology and basic and applied research. The model we suggest includes existing conceptual and empirical findings in the field of BE. While nudging theorists support the importance of studying biases and cognitive errors, RFT explains these concepts in terms of arbitrarily applicable relational responding (AARR).

The following two sections of this article address behavioral economic and behavioral analytic interpretations of choice. In the fourth section, we illustrate how RFT is able to explain and measure this knowledge with a discussion of rule-governed behavior in light of an RFT-informed interpretation of biases. The fifth section emphasizes how the importance of measuring implicit cognition is a fundamental step to understand and modify relational framing by planning scientifically sound interventions. We suggest adopting the computerized procedure implicit relational assessment procedure (IRAP) to analyze implicit cognition and its relation to human behavior. In the last section, we conclude with a synthesis that builds toward a rounder model of cognition and error.

## Nudging: Definition and Context Dependency

**Figure 1:**
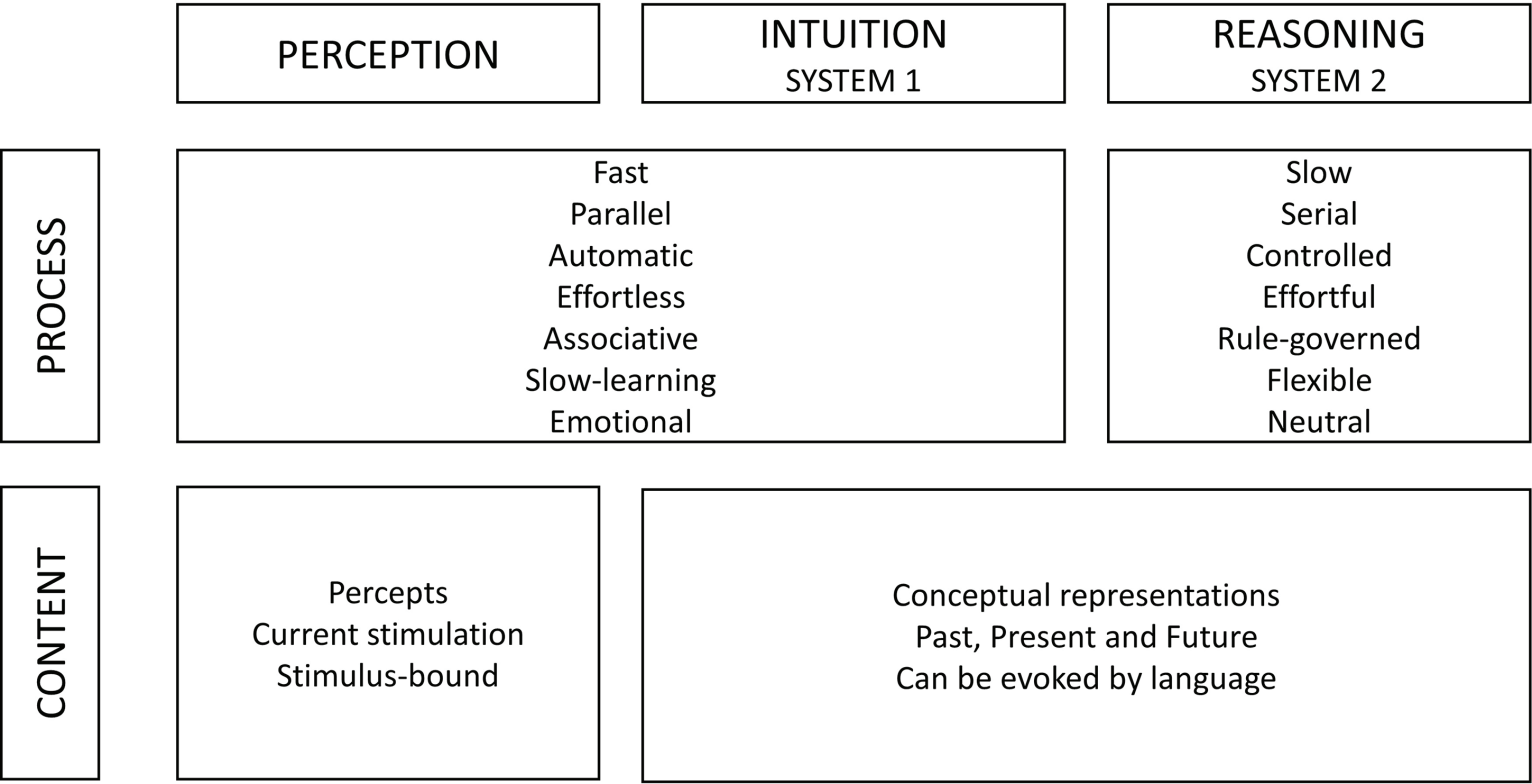
Process and content in two cognitive systems. Reprinted from Kahneman (2003, p. 698), permission not required.

The practice of nudging is commonly grounded in the research findings of Daniel Kahneman, who refined the distinction between intuition and reasoning (e.g., see Hogarth, 2001; Myers, 2002). Kahneman proposed a dual-process theory that distinguishes between a fast thinking system and a slow thinking system, respectively, denominated Systems 1 and 2 (Kahneman, 2003; see also Kahneman, 1973, 2011), and based on the original formulation of Stanovich and West (2000). Figure 1 provides a visual summary of the main attributes of intuition and reasoning, applied to process and content. Together with Amos Tversky, Kahneman pioneered the research contributions to the emergent field of BE (e.g., Kahneman and Tversky, 1972, 1979) and laid the foundations of a theory for interpreting cognitive biases (see also, Tversky and Kahneman, 1974; Kahneman et al., 1982).

Biases are systematic (i.e., non-random) behaviors that deviate from a formally correct or normatively desirable appraisal. According to the heuristic and bias programs, a cognitive bias may be defined in economic terms as a systematic error in human processing capacity, due to external influences or misattributions, based on previous concepts or experiences. Within this vision, biases are displays of humans’ bounded rationality. Conversely, the Olympian model (Simon, 1983) of rational choice theory does not program for any systematic or random error. Theories of bounded rationality represent a descriptively more adequate model than rational choice theories, according to which agents are depicted by the Olympian model or the archetype of Homo economicus (see Yamagishi et al., 2014). For example, bounded rationality may imply resorting to the availability heuristic, which intends the estimation of frequencies or probabilities based on the ease to which they “come to mind” under conditions of uncertainty (Tversky and Kahneman, 1973). More recently, Kahneman introduced the concept of *noise* “as a source of error in human decision-making. Noise, as opposed to biases, is not the systematic error of judgement but rather a randomly occurring inaccuracy, influenced by unstable situational factors” (Ruggeri et al., 2019, p. 67).

The term *heuristic* derives from the Ancient Greek meaning “to discover,” which “differs from approaches that define heuristics as rules of thumbs or as irrational shortcuts that result in decision biases” (Reimer and Rieskamp, 2007, p. 347). Albeit addressed differently according to the programs of bounded rationality and ecological rationality, respectively, heuristics represent actual explanations of the mechanisms that may lead to biases (Tversky and Kahneman, 1974); however, it has been argued that they “are too vague to count as explanations” (Gigerenzer, 1996, p. 593). Heuristics were first represented as imperfect tools of the mind that may cause systematic errors in decision making (Tversky and Kahneman, 1974). However, heuristics can be strategically employed to make a non-accurate but better-than-average judgment. For example, a possible strategy for overcoming the manifestation of potential biases in our everyday decision making is judging the probability that an object or an event A belongs to class B, based on the representativeness of A toward B (i.e., representativeness heuristic; Kahneman and Tversky, 1972; see also Samson, 2019).

Fast and frugal heuristics are a subtype of heuristics that refer to simple and task-specific strategies within the agent’s repertoire (Gigerenzer et al., 1999), such as estimating the number of inhabitants of two American cities, one of which is not familiar to the agent from before (Goldstein and Gigerenzer, 2002). Thus, whereas the heuristics-and-biases tradition has mainly been concerned with studying how and when heuristics arrive at inaccurate judgments in the sense of not being absolutely accurate, the “simple-heuristics” tradition has been concerned with studying how and when judgments can be relatively improved, even if not arriving at completely accurate judgments in the strictest sense. In the RFT domain, as we will discuss later, heuristics are responses under the control of networks related to other networks that may or may not influence the behavior of an individual.

The term *nudge*, as an operationally defined behavior modification tool, was first introduced by Wilk (1999) and popularized in the volume *Nudge: Improving Decisions about Health, Wealth, and Happiness* (Thaler and Sunstein, 2008). Thaler is considered among the founders of BE, thanks to his contributions to improving retirement savings by means of soft policymaking (Thaler and Benartzi, 2004; see also Thaler, 2018), in the forms of corrective and ethical nudges^1^. The politics of libertarian paternalism researched by Sunstein served as the philosophical precursor of the more agile term nudge (Thaler and Sunstein, 2003), which may lie somewhere on the continuum between the poles of libertarianism and paternalism.

A nudge is hence defined as any environmental manipulation meant to provide alternatives and “better” options to our System 1 (Kahneman, 2011), which is automatic, reactive, limited in processing capacities, and therefore prone to biases. Whether the agent chooses a better option is a function of adjusting its *intuitive judgment* to a better one, still under control of implicit System 1. Hertwig and Grüne-Yanoff (2017) intend nudges possessing this characteristic as forms of paternalistic learning. Nudges work by providing contextual features (i.e., a change of the choice architecture) that take advantage of the human tendency to implement an automatic thinking process (e.g., a heuristic) but differ from a biased-induced heuristic insofar as it leads people to make (presumably) good decisions. Depending on the type of intervention and whether it invites to an analysis of the agent’s own behavior, nudges may not be transparent and readily recognizable by the agent (cf. Sunstein, 2015). In fact, they may work specifically because the “recipient” needs not be aware of them. For example, Kallbekken and Sælen (2013) recorded a 20% decrease in food waste among restaurant customers, thanks to implementing two independent nudges. The first nudge included a message of social approval for multiple servings at the buffet (i.e., a transparent nudge), and the second nudge comprised replacing the old plates with smaller ones (i.e., 3 cm reduction in diameter, hence a less transparent nudge).

However, nudges may also work by bringing choice to the attention of the explicit System 2 (Rachlin, 2015), which is deliberative, reflective, and can rely on more resources and time. Examples of nudges possessing this characteristic include adding a delay prior to the moment of choice between two alternatives in an experimental session (Rachlin and Green, 1972), or signing declaration forms at the beginning, instead of at the end of the document (Shu et al., 2012).

It may be debated what the better “belly reaction” refers to, depending on the designer of the manipulation or the choice architect. Some authors debate whether a choice architect needs exist at all (e.g., Arno and Thomas, 2016, p. 2). Others distinguish between designing natural and artificial contingencies, inasmuch as the former needs not be actively manipulated (Simon and Tagliabue, 2018). Furthermore, any conflict of interests should be transparently stated: understanding whose interests the designer serves is an ongoing ethical concern. For example, the ends of the choice architect employed in a sales company who is supposedly incentivized by performance-based economic targets might be diametrically different from the ends of a choice architect who volunteers in a humanitarian organization. Nonetheless, their means might be the same. These and other issues that have been addressed by different scholars include the ethics of nudging (e.g., Sunstein, 2015), the effectiveness and the universality of nudging interventions concerning large groups or different populations (e.g., Loewenstein and Chater, 2017), and the sustainability of nudges in the long term (e.g., Tagliabue et al., 2017). What happens once the experimenters leave the research field, or the stimuli prompting behavioral change start “fading” in the background and choosers remit to old behaviors (Sunstein, 2017), needs yet be empirically addressed.

Grüne-Yanoff and Hertwig (2016) and Hertwig and Grüne-Yanoff (2017) distinguish between nudges and boosts. While nudges rest on the heuristic and bias programs, boosts rest on the simple heuristic research program (Gigerenzer et al., 1999). The authors suggest the consideration of boosts as learning tools in the context of policymaking and a way to empowering agents throughout different situations. Nudges, on the other hand, are simple and one-time-use strategies to overcome the shortages of System 1: they do not necessarily empower the agent and may on the contrary exploit its limits. For example, fast and frugal nudges are effective at providing an easy “way out” from error-prone choice behavior. In this sense, boosts represent a subset of all possible nudges, as they coincide with educative nudges (Sunstein, 2016). Hence, boosts provide a mediating cognitive component, in the form of task-specific or transversal computation capabilities.

In recurrent choice behavior, an agent may learn from being repetitively nudged toward one most desirable choice, as judged by herself (i.e., in their own best interest; Sunstein, 2018). This cognitive dichotomy sets the occasion for a further distinction, between the Planner, a fictional ideal agent bond to a System 2 information processing, and the Doer, a counter agent more prone to follow instincts and immediate gratification (Sugden, 2017). In the authors’ words, “the Planner is trying to promote your long-term welfare but must cope with the feelings, mischief and strong will of the Doer, who is exposed to the temptations that come with arousal” (Thaler and Sunstein, 2008, p. 42). The practice of nudging has been growing exponentially in academia, organizational settings, and policymaking. The application of nudges at the regulatory level of governmental policymaking cycles is especially fertile, given the advantageous cost to return rate (see Benartzi et al., 2017); this practice is usually referred to as *behavioral insights* (OECD, 2017) and aims at shaping users and consumers that look more like Planners than Doers.

From a behavior analytic perspective, nudges may be conceptualized as antecedents of target behaviors presented by the environment. They represent the setting conditions of a choice or decision and its consequent course of action. These consequences may be reinforcing or punishing but may not necessarily be intended as superimposed or artificial. Yet, the technical terms *reinforcer* and *punisher* refer to any stimulus event that follows a behavior, increasing (reinforcer) or decreasing (punisher) the probability of future occurrence of the behavior in similar conditions. Interventions can rely on either “natural” nudges, which represent any environmental aspect of the ubiquity of choice architecture, or “contrived” nudges: these intend any purposeful manipulation “to alter the environment so as to bring behavior under the control of long-term and abstract reinforcement contingencies” (Rachlin, 2015, p. 201). Hence, contrived nudges reach beyond the intuitive processing system and require the activation of System 2 to identify the aforementioned long-term and abstract reinforcement contingencies (e.g., retirement savings, weight maintenance, and environmental conservation). Whenever resorting to superimposed or contrived nudges, if possible, the intervention should be oriented at gradually fading the nudges to match the natural environmental contingencies and, hence, to maintain the durable results.

## The Behavior Analytic Model Encloses Behavior Change in the Short and Long Terms

BA represents a unified and consistent scientific approach for understanding, explaining, predicting, and manipulating both animal and human behaviors. Human behavior includes overt actions and events beneath the skin: the latter includes thoughts, feelings, and emotions. BA explains behavior in a scientific way, treating it as a variable dependent on environmental events (i.e., antecedents and consequences). The intervention aimed at manipulating the environmental factors that produce and maintain a behavior can increase or reduce the likelihood that it will occur. Hence, the analysis of behavior is useful to explain how manipulating the context may influence the choice architecture and behavior of an individual (i.e., nudging).

A widespread naïve misconception depicts behavior analysis such as ignoring consciousness, feelings, and states of mind and poses limits to its applications (Puligandla, 1974; Todd and Morris, 1983; Jensen and Burgess, 1997). However, it was made clear since the very first Skinnerian papers that a behavior analytic explanation takes into account private events (Skinner, 1945). Nevertheless, the use of technical terms, which at times may result in a distinctive jargon, has often made the rebuttals and clarifications of Skinner (e.g., Skinner, 1974) hard to understand outside the community of reference (DeBell and Harless, 1992). Understanding Skinner’s studies and contextual behavioral science perspective becomes easier if we consider that language and thoughts are behaviors, that judgments and choices are actions, and that there are no differences between implicit and explicit topographies. The experimental analysis of human cognition and emotions elaborates solutions to ameliorate the human condition and opens to the possibility of new perspectives, behaviors, and choices. Moreover, it offers an articulated and productive framework for understanding and interpreting interventions in different areas, such as psychotherapy, BE, or prosocial behavior (Hayes et al., 2001; Hayes et al., 2012; Biglan, 2015).

**Figure 2:**
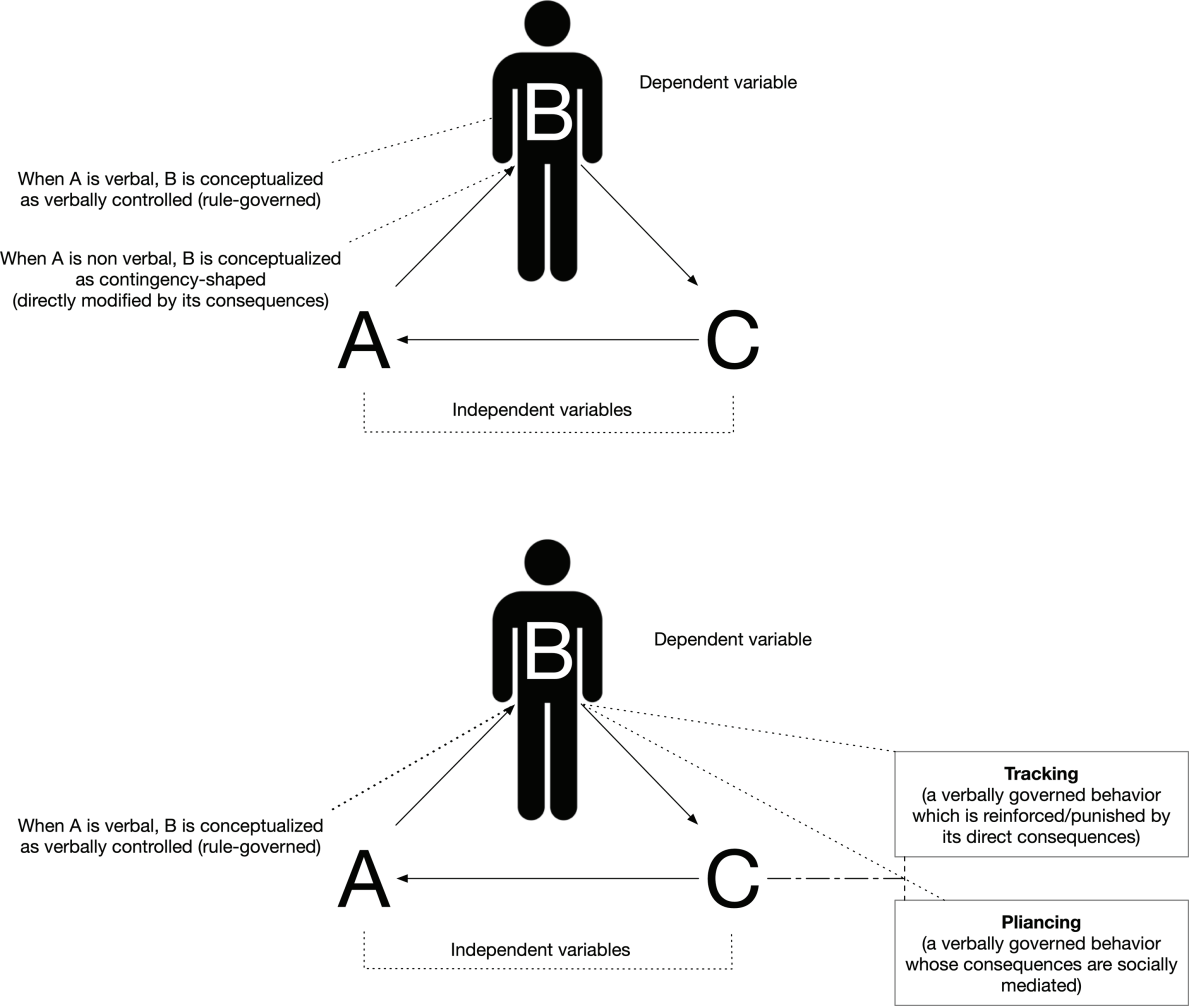
Contingency-shaped behavior and rule-governed behavior, in relation to tracking and pliancing.

The operant is considered the unit of analysis of this science and refers to direct contingencies of reinforcement or punishment, which encompass antecedent and consequences (independent variables) and modulate the occurrence and variation of behavior (dependent variable). Based on this conceptualization, Skinner (1966) offered a framework to explain the articulated differences between human and animal behavior in a seminal paper on problem solving. He distinguished between contingency-shaped behavior and rule-governed behavior. These differences are represented and further elaborated in Figure 2.

Contingency-shaped behavior is behavior established by a gradual shaping of successive approximations, such as learning to catch a ball by trial and error. Typical examples also include walking, swimming, or hitting a ball with a bat. In lay terms, they are often referred to as automatic behaviors. Thus, contingency-shaped behavior takes the definition of any type of operant behavior during which a human organism comes directly in contact with contingencies of reinforcement or punishment: these contingencies are known to modulate behavior. For example, touching a hot stove plate may be interpreted as contingency-shaped if we had experienced first-handedly the pain of touching it at least once in the past. On that one-time occurrence or in any subsequent reoccurrence, we learned that we should not engage in that behavior again, unless we are seeking pain by scald.

Alternatively, another form of human behavior takes the denomination of rule-governed behavior or verbally governed behavior, as it was subsequently renamed (Vargas, 1988). In this case, a verbally competent human organism does not need experience the direct (negative) consequences of a behavior, which are instructed, instead. In the previous example, most humans need not touch a hot stove plate in order to test whether it is indeed painful. Hence, this behavior is governed by the rule that our parents or other significant adults presented us, signaling that hot stove plates should not be touched. Most people are satisfied with such a rule, without the need of further inquiry. Shouting “It’s hot!” can halt a child close to touching a stove whether it is in reality hot, or not. Many forms of human behavior are based on verbal formulations of events and the relations between them. Some examples may include behaving under explicitly uttered instructions, such as directions, a grocery list or self-uttered instructions to solve a problem.

As with any type of mandate, warning, or prescription, verbal behavior regulates rule-governed behavior insofar as it instantiates nudging messages, which make use of language in order to be transmitted and followed. In fact, we may image a message emphasizing a social norm written in Chinese and highly impactful in a Chinese setting, to be ineffective among non-Chinese speaking receivers, without undergoing overwhelming comprehension issues. Some rules might not exert any influence on behavior for two possible reasons. First, because people have insufficient control of non-verbal contingencies; in other words, they may not have experienced a rule, or they do not have the skills to implement the behavior governed by these rules. Second, because the source is, or is perceived as, unreliable (Stewart et al., 2006).

The distinction between contingency-shaped and verbally governed behavior is not trivial. Researchers focused on the analysis of their functional properties and discovered intriguing differences that are relevant to the analysis of biases and nudging. For example, verbally controlled behavior has properties that can become drawbacks: “when behavior is controlled by verbal rules, it tends to be relatively insensitive to changes in the environment that are not contacted by or described in a rule itself” (Hayes et al., 1999, p. 28). Human beings have the capacity to pursue their verbally governed behavior in spite of aversive contingencies. We continue doing things that do not work just because “we are right,” or because “they should ultimately work,” even if the consequences of our behavior are not exactly what we are aiming at. In short, sometimes we act against our own best interests, even when we know it to be the case. In a similar way, the BE concept of hindsight bias depicts overestimating the likelihood of past experienced events (e.g., see Taleb, 2009).

**Figure 3:**
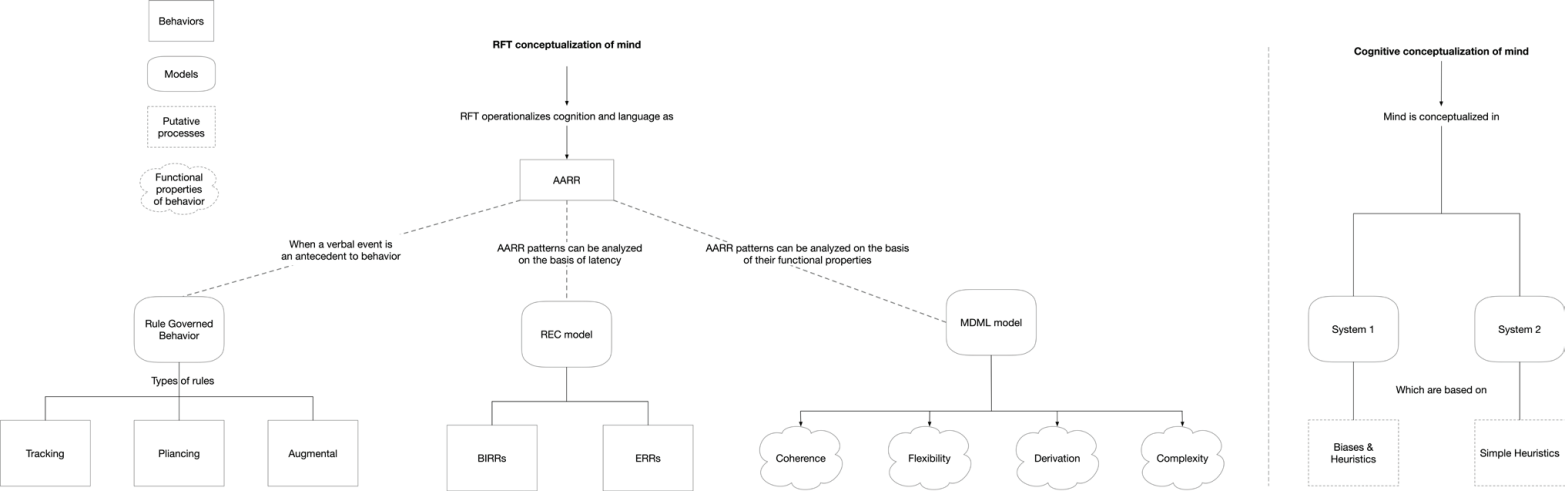
Commonalities between RFT and cognitive conceptualizations of mind.

RFT is a post-Skinnerian analysis of cognition and language, and it offers an experimental interpretation of this supposed insensitivity to contingency effect included in a more general vision of cognition and language. It comprehends a conceptualization of these properties of verbally controlled behavior that are relevant to nudging and that describe persistent patterns of non-functional behaviors influenced by cognitive biases (such as the hindsight bias). They are nested under a new contextual behavioral theory of language and cognition. RFT extends BA’s set of operant principles to analyze higher human cognitive functioning, including rapid intuitive judgment encompassed in System 1 and the conscious awareness (i.e., analytic thinking) of System 2. In this context, framing is used metaphorically to label a particular kind of human behavior, responding to arbitrary relations between stimuli under contextual control. Figure 3 represents a schematization of the two approaches, which feature the commonalities of how cognitive processes are, respectively, conceptualized. The next section is concerned with the roots of RFT, and it illustrates the conceptualization of cognitive functioning.

## Relational Frame Theory, Verbally Controlled Behavior, and Implicit Cognition

RFT is a functional and contextual explanation of human cognition and language, which are conceptualized in terms of derived arbitrary relational operant responding (Hayes, 1989; Hayes et al., 2001; Long et al., 2010; Törneke, 2010). We learn directly from experience (contingency-shaped behavior) and from relating symbols to one another (language: i.e., verbal-governed behavior). When relating occurs, the transformation of stimulus function changes the properties of symbols (e.g., nouns, sentences, and symbolic networks) such that an individual experiences attractive or aversive reactions. These reactions may be alerting or blocking at any given moment, although the agent has no direct experience with the description of the verbal utterance. In relational responding, a response is emitted as a function of one stimulus, given the presentation of another stimulus. Derived relational responding (DRR) is a class of contextually controlled responses that can be (1) either arbitrary or non-arbitrary and (2) learned through repeated exposure to multiple examples (Barnes-Holmes et al., 2001; Whelan et al., 2006; Campbell et al., 2011).

DRR is considered non-arbitrary when responding is related to the formal properties of the stimuli (e.g., color, shape, and size), for example, when an organism responds to *long/short* or *big/small* characteristics. The term *non-arbitrary* implies that this class of responses is not subject to social whim or convention. By contrast, arbitrary applicable relational responding (AARR) refers to a class of responses that are controlled by part of the context that specifies the relation between stimuli. The relational response can be brought to bear on any relation, independently of their formal non-arbitrary properties. A child asking its parent to watch the “big soccer match” together is an example of AARR. Whatever the ending score of the game, the child is able to utter that it was the final that indeed made the soccer match *big*. In this hypothetical scenario, RFT suggests that the exposure to multiple exemplars of *big/small* (e.g., boxes, shoes, and bottles) brings the relational response to an abstract point that it can be applied under conditions in which there is no formal relation to the original characteristic, as in the case of a soccer match.

AARR is defined by three functional properties: mutual entailment, combinatorial entailment, and transformation of stimulus functions. One aspect of derived relations is that they are mutually entailing. In general terms, this relation may be described as: if A → B, then B → A. For example, if an arbitrary relation is learned between two terms, such as computer is related to pangu, then pangu becomes related to computer. In the simplest case, a human being can respond to computer → pangu as a frame of coordination (i.e., a relation of sameness). However, mutually entailed relations are not always based on identity. For example, computer can be bigger than pangu, which entails that pangu is smaller than computer; or pangu can come before a computer, which entails that computer comes after pangu. It should be noted that both these relations and their corresponding sounds are arbitrary, independently from the stimulus to which they refer. In turn, this is also an arbitrary relation: we may switch the object-sound relation, calling a computer pangu, and vice versa. Moreover, a verbal community (e.g., the readers of this paper) may reinforce the understanding and use of this inverted relation.

Combinatorial entailment includes three or more stimuli and depicts the capacity of relational responses to combine. In the preceding paragraph, computer has been related to pangu. If we now relate pangu to Λ, we derive that computer → Λ, and Λ → computer, without any further training. If these relations are established within a frame of coordination, we respond to computer, pangu, and Λ as similar stimuli. Furthermore, we can arbitrarily relate computer, pangu, and Λ in terms of bigger and smaller. By asserting that computer is smaller than pangu, and pangu is smaller than Λ, we derive that Λ is the biggest of the three terms.

The transformation of stimulus functions is a crucial consequence of mutual entailment and combinatorial entailment. Let us imagine a young child who is not acquainted with the value of money being asked to choose between a €0.50 coin and a €1 coin to buy an ice cream. Whether the child’s choice is between receiving the big or the small coin, the physically bigger coin (i.e., €0.50) is supposedly preferred to the smaller coin (i.e., €1). However, if the child is exposed to the fact that the smaller coin can buy a bigger ice cream at the store (e.g., by looking at what another child is buying), the child can derive and choose the smaller coin the next time and thus receive the bigger ice cream. Coin values are arbitrarily related to their dimensions, and so is their function. The economic value of a coin is one of the possible stimulus functions of this object.

The transformation of stimulus functions expands the possibility of a human being with respect to all environmental interactions. It may also help anticipate events by bringing relevant stimulus functions on arbitrary symbols, such as words. For example, Vervoort et al. (2014) trained two four-member classes of abstract figures with a matching-to-sample procedure, in which a sample stimulus and two or more comparison stimuli were presented. Choices of correct arbitrary matching between sample and comparison were reinforced during training, while incorrect responses were ignored. This is the basic procedure for learning to relate A to B and B to C and derive C to A, A to C, B to A, and C to B (Sidman and Tailby, 1982). In the study of Vervoort et al. (2014), after the surfacing of the derived relations, only one item of a stimulus class was conditioned as an aversive stimulus, pairing it with a mild electrical shock. It followed that all other stimuli belonging to that class acquired the same function without any additional training. Skin conductance and shock expectancy rating were measured. A conditioned and generalized response of fear was derived by transformation of stimulus function, from one to all members of the symbolic class. This happened without any direct training of the other three stimuli belonging to the class, as part of the first training. Moreover, no reaction was registered in the presence of any of the items of a second unconditioned class (for an analysis of symbolic fear and avoidance generalization and its role in human behavior, see also Dymond and Roche, 2009; Dymond et al., 2015). These areas of research in symbolic networks show that psychological effects can occur without direct learning. These effects may be partly attributed to the symbolic processing of Systems 1 and 2.

Relations of coordination or sameness such as the one in Vervoort et al. (2014) are not the only frames and under which humans learn to respond. A number of other relational frames have been identified, including difference, oppositeness, comparison, if … then, before … after, and so on. We are not only able to respond to bidirectional links between events but also able to respond to relations and relational networks (Barnes-Holmes et al., 2017), which add complexity and modify the flexibility of derived responses. Combinatorial entailment points out the degree of complexity of networks of relations and the degree of unpredictability of derived psychological effects. Various degrees of complexity in DRR have been empirically investigated, such as the different ways in which derived relational response models may differ in terms of their properties (e.g., number of stimuli, relation, and varieties of contextual cues). Some of the properties that are included in a bigger construct like Systems 1 and 2 may be further operationalized and give access to more effective nudging practices. Specifically, relational responding is context-sensitive and context-driven.

### The Selective Role of the Context

The context selects situationally relevant psychological functions at any given time. The underlying derived relation transforms the function of a stimulus whenever relational networks are related to one another. The word *pangu* introduced in the previous section was suggested by one of the authors as a possible arbitrary sound to be related to the word *computer*. However, after a Google search, we realized that Pangu is the first living being and a God creator of the universe, according to some versions of Chinese mythology. Providing this verbal context now offers the reader the opportunity to relate computer and pangu in different ways. For example, computer can be part of a network in which other stimuli are other mythological Gods, such as Zeus, Jupiter, or Odin. Notwithstanding, according to further Google search results, Pangu is a type of software. With this newly acquired information, the function of relations in our example can be further transformed in novel and unpredictable ways. However arbitrary and non-sensical this example may be, it provides a basic understanding of how RFT conceptualizes relations as arbitrary responses, which are contextualized within a more sophisticated model in the following section. But first, let us further elaborate on the previous example relating pangu and computer and refer to the learning histories of two hypothetical but plausible groups of readers of this work.

Supposedly, each of the two groups of readers has different learning histories with respect to Chinese mythology and computer science, up to the point where the term pangu was introduced. If we asked the members of each group to define pangu up to that point, we would have probably recorded answers influenced by their learning histories. If we flipped their learning histories, devising an experiment that associated pangu to software among the subjects knowledgeable in Chinese mythology, and vice versa among the computer geeks, we would probably have recorded contrasting responses based on how strong each group member’s history of learning with respect to software or mythology respectively is. These verbal relations are naturally established in the community where Chinese or computer geeks live. DRR for pangu → software is likely to occur faster among computer geeks than among Chinese mythology experts, and vice versa. The discrepancy between naturally learned verbal relations and specific laboratory-induced relations has been a paradigm to study and offers a new operationally sound way to conceptualize implicit cognition, which is thought to be related to choice (Hughes et al., 2011).

Watt et al. (1991) investigated this discrepancy by studying religious biases in Northern Irish Catholic subjects and Northern Irish and English Protestant subjects. The authors conducted a very simple experiment, which aimed to understand the effect of clashing the verbal history of learning of an individual with an opposite history of learning provided in the laboratory. Instead of nonsense syllables or other unknown stimuli useful to by-pass the learning history of the subjects, students were purposely trained to match Catholic names with nonsense syllables, and those syllables with Protestant symbols. The derivation test was built in such a way that subjects could respond according to either their social learning history (i.e., matching a Catholic name with a Catholic symbol) or the experimentally generated equivalence relations (i.e., matching a Catholic name with a Protestant symbol). The students enrolled in this research represented different histories of learning with respect to the stimuli used.

After the training phase and the derivation phase connected to the experimental task, two additional tests were presented. Three options were presented for each test: the first included two Catholic and one Protestant names; the second included one Catholic name that was not previously included in the training, one Protestant name, and one neutral name. More than half Protestant and Catholic Northern Irish students consistently chose a Protestant name in the presence of a Protestant symbol in all three derivation tests. All English Protestant and the remaining North Irish Catholic students chose the Catholic name in the first test, with a high degree of variability in the responses to the other alternative tests. These findings suggest that learning histories represent a relevant variable that affects how new relations are derived.

This model represents a way to study the influence of the agent’s previous learning history on the conditions in which choices are made. In other terms, it simulates how information is related. Barnes-Holmes and peers elaborated further on this scenario and proposed the relational elaboration and coherence (REC) model. The REC model is a RFT-based account of cognition that explains the formation and retention of opinions and beliefs, including choice, prejudice, and biases (Barnes-Holmes et al., 2010a; Hughes and Barnes-Holmes, 2013). These behaviors are occurrences of DRR that can either be brief and immediate or extended and elaborated. Brief immediate relational responding (BIRR) and extended and elaborated relational responding (EERR) are operants that can interact with each other; they represent the same process of relational framing. The difference between them is the time elapsed between the stimulating event and the response. Thus, they reflect the same behavioral process under two different settings, which are defined by the presence or absence of time pressure to respond.

BIRR refers to responses that occur within a few seconds from the event; EERR indicates responses that occur after longer periods from the antecedents (e.g., Barnes-Holmes et al., 2010b). According to other conceptual models, BIRR is often termed as automatic and implicit cognition and related to the presence or absence of specific cognitive processes such as awareness or attention. Functional contextualism denotes the epistemological vision in which RFT is rooted. As it rejects any mental mediating process, we use the terms brief, immediate, extended, and elaborated as simple descriptors of the occurrence of behavioral phenomena.

Experimental procedures that are designed to target rapid responses, such as speed categorization tasks [e.g., implicit association test (IAT)], should be influenced to a lesser degree by extended and elaborated relational responding. By contrast, when compiling questionnaires during focus groups or interviews, where time-constraint is absent, the REC model submits that the observed behavioral patterns reflect more extended and elaborated responding.

RFT claims that AARR is the operant class behind human verbal behavior and cognition. Yet, the process of derivation and consequent transformation of stimulus functions does not always lead to correct evaluations. As long as we look at the bi-directionality of mutually entailed relations, both of them maintain the same level of precision. However, when relations are combined, precision may be loose and unspecified. According to our previous example, if computer is different from pangu, and Λ is different from pangu, we have no way of knowing what relation exists between computer and Λ.

### Dealing With Uncertainty: Coherence, Prejudice, and Biases

Humans show difficulties in dealing with uncertainty, to the point where it might also be related to psychopathological conditions (e.g., Reuman et al., 2017). In the presence of arbitrary related events, networks, and networks of networks, verbally competent humans show a tendency to maintain coherence. Although relational responding is arbitrary (e.g., pangu and computer may be arbitrarily switched with reference to a God and a software), the verbal communities of reference usually reinforce and coherently maintain the relations between them. The same may be asserted regarding the structure of a sentence, in which rules of coherence are summarized in what it is usually referred to as grammar, or with what we term as meaning. For example, the coherence of the relation between the sound *burro* with a donkey or butter is differentially reinforced in the Spanish or Italian speaking communities, respectively.

*Coherence* is socially reinforced since a child’s first exposure to learning any language. Coherence and experiencing the consistency of events and facts become generalized reinforcers for verbally competent humans (Healy et al., 2000; Blackledge et al., 2009). When humans need to describe inconsistencies in their experiences, they verbally resort to building coherence between events, even in the presence of a threat of paying the high cost of psychological suffering (Prevedini et al., 2011; Villatte et al., 2015). Hence, coherence is reinforcing; uncertainty, which represents a state of relational incoherence, is punitive. An Italian speaker who has little or no knowledge of Spanish and asks for butter in Almería (Spain) using the Italian word “burro” might be faced with a number of reactions. Most of them are likely to possess an abolishing function, which will reduce the chance to use the word “burro” in the future in that language context. Coherence might enter in different topographies of human AARR. For example, trusting a well-mannered stranger on the basis of previous positive interactions, which may be behavior prone to bias; or choosing to buy a new product of a specific brand, following a history of purchases of other products of the same brand (i.e., brand fidelity). Biases and decision making are reinforced by the coherence of self-narrations. Moreover, the REC model affirms that humans need coherence: they are unlikely to change their evaluation merely by the simple instruction of “thinking the opposite” (Barnes-Holmes et al., 2010a).

A further element of a functional analysis of uncertainty maintains that BIRR and EERR may interact. Consider, for example, an Italian undergraduate student who lives in a foreign country and is faced with the decision of sharing accommodation with another student, either of the same ethnicity or a different one. According to his or her verbal relational history, the first DRR (BIRR) might be a stereotyped prejudicial evaluation. However, a more elaborate DRR (EERR) may imply a different decision by assigning less importance to the ethnic characteristic and more to the opportunity of exploring new and different customs and traditions (Roche et al., 2002). This latter choice may be considered an EERR, for it is not automatic and requires a longer processing time than the prejudicial choice. Hence, BIRR and EERR differ based on the number of possibilities that may be evaluated at any given time, and on the time required, regardless of the content of the final choice. Whichever function is established depends on the influence of both verbal and social contexts. Arbitrarily established relations, in turn, transform stimulus functions of attractive or aversive nature. If the student is told that a number of typhoid cases have been registered among foreign students, welcoming the new roommate might be aversive. Thus, whenever environmental control by nonverbal stimuli (e.g., the sight of a potential disease), are weak or absent, humans turn to verbal descriptions (rules) to steer their behavior. In this case, the potential risk of infection is evaluated and contrasted with the opportunity to know new people and traditions. The student may consider the base rates and probability of contagion, obtain more information about the cases of typhoid fever, or consider the importance of welcoming diversity. In this example, the student continuously relates networks of relations to other networks. New rules are elaborated from the agent’s own previous rules or others’ rules, which may or may not be followed in future occurrences (see also Skinner, 1966).

It is possible that BIRR and EERR are inconsistent with each other, and additional relational responding may be needed to reduce or resolve this inconsistency. According to the REC model, BIRR and EERR are able to interpret the interaction between levels of functionality of the relational repertoire. However, they do not capture all aspects of the interaction between the organism and the environment. The REC model is an account of derivation and complexity of relational responses, but it does not consider the coherence with which these relational responses remain part of the individual repertoire (Barnes-Holmes et al., 2017); nor does it consider the flexibility that allows these responses to change in relation to context (e.g., O’Toole and Barnes-Holmes, 2009; Barbero-Rubio et al., 2016). Consequently, Barnes-Holmes et al. (2017) proposed the multi-dimensional multi-level model, which includes four dimensions (complexity, coherence, derivation, and flexibility) and five levels of relational responses. Relational complexity refers to the stimuli involved in the relationship, which may involve simple stimuli or other relationships. Coherence refers to how uniform the relational response is compared to the agent’s previous experience of interacting with the environment (i.e., learning history). Derivation refers to the untrained or not specifically learned behavior of responding to relationships between stimuli. The longer the time the relational response is repeated for, and reinforcement is provided by the reference community, the lower the level of derivation is: this signifies that the repertoire of responses is more consolidated. It follows that quicker responses in an IRAP task signal more coherence with this history. Furthermore, *flexibility* refers to the possibility of modifying a relational response as a function of a contextual variable. Hence, it is possible to nudge a response acting on some element of the context in a direction, which might be less practiced by the individual.

For example, a student is standing in front of a drink dispenser and is presented with the choice between one of the two alternative drinks: Soda A and Soda B. The student has had previous experience with both drinks, whereas his or her family used to buy Soda B (high coherence and low derivation) and his or her friends rank Soda B as good as Cola A, which is better than Soda A (low complexity). It follows that the student would presumably choose Soda B. However, as he/she is approached by a colleague who asks his/her to drink a Soda A together, his/her choice has now been updated between Soda A (high flexibility) and Soda B (low flexibility). The colleague’s request works as a nudge: it represents a contextual element meant to direct the agent’s attention toward certain aspects of its encompassing environment, instead of other aspects. This example illustrates that the multi-dimensional multi-level model is able to explain choice behavior that may further be measured with an IRAP task. Hence, IRAP response options can be simply considered as indicators of relational coherence, for the task does not measure implicit cognitions *tout-court*. Instead, it measures a relation between the stimuli and the influence of the subject’s learning history, whenever responding to a relation of opposition (Barnes-Holmes et al., 2018).

### Verbal Rules: Pliancing, Tracking, and Augmental

RFT has been developed to answer the following question raised by Hayes et al. (2002), which might sound oddly familiar to any specialist in BE: “Under what conditions do people select among available rules or generate new ones, follow rules when they are available even though they conflict with other sources of behavioral control, and change them when they no longer work?” (p. 100).

According to RFT, any antecedent possessing symbolic properties, including nudging tools that may be comprehended in this category, are to be considered *verbal*. A verbal antecedent alters human behavior through the transformation of stimulus functions that are the result of the contact with the elements stated in the rule. In the example above, the rule could take the form of “Would you like to drink Cola A with me?” Verbally governed behaviors interact with contingencies of reinforcement, which can influence its functional properties, namely, flexibility. Based on the consequences of the action promoted by the rule, RFT classifies verbally governed behavior in pliancing and tracking rules. They are based on an empirically functional distinction (Törneke, 2010).

Pliancing is human behavior that follows a verbal utterance and is reinforced or punished by socially mediated consequences. A child who wears a coat after its parent referred to the bad weather outside and receives approval for that behavior is an example of pliancing. Tracking is human behavior that follows a verbal utterance and is reinforced or punished by the direct consequences of the act described in the rule. Following the manufacturer’s instructions manual on how to operate a new mobile phone is an example of tracking: the user receives feedback directly from the operations made on the mobile. In both pliancing and tracking, relational networks put a human being in contact with two sets of different contingencies: (1) socially mediated contingencies and (2) direct contingencies.

Once pliancing repertoires are acquired, they become general operants, and there is no need for reinforcement. Ultimately, we follow plies because we learned to follow them. Pliancing can offer advantages that have supposedly played a crucial role in the selection and evolution of the human species, insofar as it may have overridden the effects of direct contingencies. In other words, we are able to avoid dangers by following the advice of someone we trust, without the need of testing whether it is right or wrong. We want a child to stop at the utterance of the words “It’s hot!” preventing it from touching a hot stove, or in the presence of other contexts bearing risk. A sign displaying “Danger: electrical hazard!” should hardly be tested at the expenses of one’s life. We learn pliancing whenever we follow our parents’ advice, which is supposedly meant to prevent harm and danger. Over time, socially mediated contingencies reinforced our past behavior for following rules or punished it for not following them. Hence, pliancing helps us to avoid dangers. Nevertheless, a person who tries to avoid presumed or real punishing contingencies (e.g., behaving in ways that make others happy) may develop a repertoire that prevents him or her to coming in contact with other contingencies than those provided by and through others. This might be the case of attending classical music concerts because that is “what intellectual people should do,” or in order to merely please a friend or partner. When this pattern of behavior predominates in the agent’s repertoire, it may hinder tracking, thus reducing behavioral flexibility and increasing rigid behaviors and vicious circles.

A third set of rules is called *augmental*: differently from the first two types of rules that specify contingencies, augmental rules alter the reinforcing properties of the consequences specified in the rule. This type of relational network puts a behavior in contact with its consequences, changing the “strength” of that consequence. For example, several virtuous behaviors may be the result of the following augmental rules: protecting the environment, conserving the energy conservation, and in general leaving a better world for future generations are forms of environmentally sustainable augmental rules. Humans can act the following rules that specify contingencies that may occur even after their death, without having to come in contact with them during their lifetime. Thus, we can learn to stand for what is important to us in our lives and alter the value of difficult or even punishing contingencies (Törneke et al., 2008).

In summary, according to a functional-contextual behavioral vision, human behavior is conceptualized as being under the control of a set of variables (namely, antecedents and consequences). Some of them are verbal and may be referred to as *cognitive* variables: they can produce a wide range of functional characteristics of human behavior and, in specific circumstances, have been named biases. They vary the functional properties of human behavior along dimensions such as derivation, complexity, coherence, and flexibility. These sets of verbal antecedents come from two kinds of learning histories, which may have been shaped by direct or socially mediated contingencies. These contingencies produce different functional effects because of the different proprieties of the developed relational responses. Another set of verbal variables may change the way agents evaluate direct or socially mediated consequences of behavior, by altering their reinforcing or punishing function. Nudges interact as additional contextual independent variables in the set of contingencies. They orient human behavior and possibly modify the functional properties of the relevant verbal or nonverbal variable. Thus, nudges can modify the context where relational responding occurs, generating different transformations of stimulus functions (meaning) and re-orienting human attention to other cues of the environment that evoke different responses than those originally programmed according to history of the subject (Barnes-Holmes et al., in press). Conversely, conditions that do not feature any corrective or educative nudge may influence choice behavior according to the learning history in a “biased” way. This operationalization of the practice of nudging, as well as including it in a behavioral model, may help better understand the interaction among single behavioral components, which include their underlying cognitive ones. The effects of both behavioral and cognitive components are understood in terms of their function in the contingency and may be altered accordingly for increasing agents’ effectiveness toward biases. According to the standpoint herein put forward, biases are not “mistakes” or alterations in the functioning of the human mind, but the natural and physiological instantiation of human behavior given a history of learning and derivation under the given circumstances.

## Studying Cognitive Biases

### Implicit Relational Assessment Procedure: A Measure of Bias Prior to Planning Interventions

The empirically based distinction described in the previous section may help to better understand cognitive biases and heuristics, which are considered DRR in the context of environmental events. It may also improve the operational definition of behavioral repertoires that BE aims to address. RFT offers an understanding of how verbal and cognitive events are based on relational responding. Further questions that RFT addresses include: (1) How is AARR learned? How do networks and their functional properties develop? (2) Are rules learned or self-generated? (3) How may relational responding influence behavior in terms of its functional properties? In partial answer to the last question, some authors studied the experimental conditions under which BIRR is evoked and measured, in contrast to EERR. Barnes-Holmes et al. (2006) elaborated the IRAP as a measure of cognition in terms of relational behavior, instead of associative activity. In other words, IRAP investigates a more System 1-dominated process. Furthermore, IRAP seems to offer better specificity to what is observed and measured than other implicit cognition tasks do, such as the IAT. To give a trivial example, let us say that we are interested in investigating the relation cats → dogs. When we mention that two animals, a cat and a dog, are associated, we generally do not specify their association. In the context of mammals, they can be considered similar, because they both breastfeed their whelps. Conversely, in the context of the popular saying “fighting like cats and dogs,” the two are related to a frame of opposition. Another particularly relevant difference to the field of BE between IAT and IRAP maintains that the IAT can only assess biases determined by comparing two different combinations of stimuli (relative stimuli); notwithstanding, the IRAP can assess non-relative biases. In fact, measured latencies are relative to two sets of relational responding, one which is conditional to social and cultural conventions, and the other is specifically designed to counterbalance those conventions in the experimental conditions.

The IRAP is a computer-based task, which features the presentation of one sample stimulus, one or more comparison stimuli, and specific relational terms. These are contextual cues (e.g., *similar, opposite, more,* and *less*) that allow the measurement of relational responding between task stimuli (Barnes-Holmes et al., 2010a). The response speed of the task required by the IRAP is a measure of the participant’s previous learning history, combined with the contextual cues proposed by the IRAP (Barnes-Holmes et al., 2006). IRAP compares the result of a subject’s interaction with environmental events that have conditioned his or her learning history with the learning history proposed by the experimental task. The latter reflects how the experimenter thinks the group of participants responds to the research. The IRAP compares responses under two main conditions: consistent (bias-consistent task) and inconsistent (bias-inconsistent task) with the learning history of the subject and his pre-experimentally established verbal relations. The rationale for IRAP is that the reaction response is faster (BIRR) in the tests consistent with the subject private verbal relations or beliefs and relatively slower in the inconsistent conditions. For example, a faster response should be recorded in the bias-consistent task “love and pleasure, if similar,” when compared to the bias-inconsistent task “love and pleasure, if opposite.” D scores are indicators of the hypothesized discrepancies in the experimental conditions (Barnes-Holmes et al., 2010a). Among others, the IRAP has been used to investigate biases, prejudice, psychopathologies, perspective taking, positive, and negative expectancies on future events, among others (Golijani-Moghaddam et al., 2013; Vahey et al., 2015).

### Addressing Consumer Behavior Through the Implicit Relational Assessment Procedure

Although the IRAP is mainly a choice-based task, a limited number of studies have focused on questions relevant to BE thus far. In a decision-making task, Modica et al. (2017) used the IRAP to analyze purchasing attitudes contrasting taste and affordable pricing. The authors presented two relational response options (true/false) in the context of six words related to “enjoyable taste,” six words related to “affordable-priced food,” and two targets: like and dislike. The different combinations of samples and targets within the experimental procedure were distributed in four trial types: tasty-like; tasty-dislike; cheap-like; and cheap-dislike. In the consistent blocks, participants were instructed to respond according to the hypothesis of purchasing a product based on its organoleptic characteristics, rather than its price. Although the researchers assumed that the participants would respond as if taste determined the purchase of food more quickly than price would, the participants responded in the same way to the two tests of the IRAP task (consistent and inconsistent). In other words, contrary to the experimental hypothesis, the results showed that the participants’ implicit attitudes toward taste and affordable pricing have the same value.

More recently, Rafacz et al. (2019) used the IRAP to investigate the effects of motivational (augmental) assertions on individual versus cooperative responding under two different pay-for-performance contingencies. Data showed that both motivational and financial contingencies had an effect on performance. However, motivational statements affected the way participants chose to behave cooperatively under economically neutral conditions. Research resorting to the IRAP has indicated that BIRR may represent a better predictor of behavioral outcomes than EERR.

In a different setting, IRAP and explicit measures of cocaine craving were used to assess 25 cocaine-dependent participants before and after a 6-month-long outpatient treatment program. Results showed that poorer treatment outcomes were associated with stronger implicit beliefs about the positive effect of cocaine use during baseline. Moreover, no correlation was found when the same beliefs were measured with self-reported instruments (Carpenter et al., 2012).

### Brief Immediate Relational Responding and Extended and Elaborated Relational Responding as Forms of Nudging Systems 1 and 2

Albeit limited in number, the studies included above suffice to testify the potential of adopting the IRAP procedure in BE research. The domain of consumer behavior has been traditionally dominated by nudging techniques, even though they were referred to as placement, marketing strategies, or incentives, with immediate practical implications. Thus, a nudging intervention meant to promote the sale of healthier food products (e.g., the first example reported in the previous section) requires the activation of the more “thoughtful” and effortful System 2. This involves breaking the current relations of responding and promotes new derivations that encompass origin or caloric content. The second example reported in the previous section confirms the assumption that we may increase performance with memos or speeches in an organization. Furthermore, aligning it with financial contingencies may maximize performance. The third example above offers a way to investigate and predict outcomes as a function of the strength of BIRR (i.e., “gut feelings”) and their coherence and flexibility in influencing consumer behavior. It opens to new ways of investigating why nudges could fail (c.f. Sunstein, 2017) and how to modulate nudging interventions with different types of populations, based on their learning histories and functional properties of relevant verbal variables. All three examples represent a basis for nudging interventions, not necessarily of the virtuous type, yet legitimate. They possess the property of empowering the choosing agent by widening its set of choice and allowing for reinforcing consequences apt to maintaining future occurrences of behavior.

We submit that BIRR and EERR may address processes that are included in the dual-process theory represented by Systems 1 and 2 (Kahneman, 2011). Similarly, learning history, coherence, and relational responding may be interpreted to address the processes targeted by the associatively coherent model described by Morewedge and Kahneman (2010). However, there are deep differences in terms of epistemological roots and in the level of analysis and manipulation of the relevant independent environmental variables in basic and applied research. While the former originates from an internalizing model that is more epistemologically prone to look to unmodifiable variables by definition, the latter originates from externalizing epistemological roots. These are tightened to environmental interventions (nudging) in terms of functional links between independent variables. They are the only variables to act upon whenever addressing the problem from an internalizing epistemological point of view. Putting this into a metaphor, it is like studying a dog’s salivation in accordance with environmental factors. Dogs were known to salivate to other stimuli beyond food, long before Pavlov addressed this phenomenon. Although to date we have a deep neurological understanding of this phenomenon (e.g., Kandel, 1983), Pavlov’s account offers a way to understand how environmental factors act to evoke or extinguish it. Moreover, Pavlov’s account provides an account to manipulate the phenomenon whenever in need to ameliorate the human condition, such as in psychotherapy (e.g., Arch and Craske, 2009; Martin et al., 2010; Waters and Pine, 2016).

Moreover, conceptualizing cognition and language in terms of relational responding sheds new lights on how language may alleviate the human condition. For example, acceptance and commitment therapy are a clinical technology developed from RFT (Hayes et al., 1999; Villatte et al., 2015), and other non-clinical applications are rapidly spreading in professional and educational settings (e.g., Higbee, 2009; Flaxman and Bond, 2010; McKeel et al., 2015; Presti et al., 2017); in addition, it is used to train complex behaviors, which include social behavior (Scagnelli et al., 2017). The applications of RFT target transformation of stimulus functions, which address all aspects of human life that are quintessentially verbal, by creating new contexts for relational responding. For example, if a child verbally relates fruit and vegetables in a frame of coordination consistent to “food that I do not like,” any kind of fruit or vegetable can be refused without tasting, solely on the basis of this verbal rule. Principles from BA and RFT may inform the design of an intervention in which a daily portion of fruit and vegetables is made available to children, and superheroes motivate them to taste new foods, posing as models to be imitated. Moreover, putting a child in contact with the direct contingencies of taste may create the conditions to derive new or different relational networks. The child may associate the term *good* to fruit and vegetables and further promote augmentals on healthier eating habits. Extensive research has shown consistent increases of fruit and vegetable consumption following similar interventions (e.g., Horne et al., 2009; Presti et al., 2015).

Thus, eating behavior, cooperation, and addictive behavior are mediated by relational frames that comprise the cognitive bases on which choices are modulated and may be nudged. Verbal behavior determines to a large extent the likelihood that choices are learned and repeated over time. Verbal behavior includes the justifications we give ourselves for propending toward one or another alternative or the reinforcing effects on our social network. Given the learning history of the individual and the degree to which future choices may be indirectly “taught,” the distinction between nudges informed by relational frames and boosts may no longer be necessary to reach better-informed and longer-lasting choice behavior.

## A Consistent Model for Choice Architecture

To date, there is an extremely limited number of conceptual studies on nudging that make direct reference to BA. Nevertheless, there are instances in which the two disciplines are most likely to inform each other. Herein, we provide an account of how an analysis of cognition informed by RFT may help broaden the behavior analytic model of CBS. According to Furrebøe and Sandaker (2017), there are three aspects of similarity between BA and BE, which we endorse and extend to nudge theory: “(i) human choice behaviors (ii) how proximity in time and space between behavior and environmental events influence behavior and (iii) why many species seem to behave in ways that cannot be explained by self-interest” (p. 316). The first point features experimental work on choice behavior under concurrent schedules of reinforcement (Rachlin and Green, 1972; Rachlin, 1976; Yi and Rachlin, 2004) and encompasses the exten**s**ion of some of those findings to nudging (Rachlin, 2015).

The second point emphasizes how proximity as contingencies and schedules of reinforcement is fundamental concepts in BA that need inform the choice architectures of nudging (e.g., Furrebøe and Sandaker, 2017; Tagliabue et al., 2017; Simon and Tagliabue, 2018). The preference across species for smaller-sooner over larger-later rewards is a phenomenon of investigation in both BA and BE traditions. Examples of applications of hyperbolic discounting in BA may be found, among others, in the works of Fisher and Mazur (1997) and Furrebøe and Sandaker (2015). They provide an empirical measure of the attractiveness and control exerted by abstract and delayed reinforcers.

The third point, however, on alternatives to self-interest models, has not received as substantial contributions from BA as the former has. For example, BE has been consistently concerned with the empirical study and formulation of models of reciprocity (e.g., Camerer, 2003), fairness (e.g., Fehr and Schmidt, 1999), and altruism (e.g., Sobel, 2005). Although altruism is complex phenomenon and has been addressed in several studies within BA (e.g., Rachlin, 2002, 2016; Rachlin and Locey, 2011), it represents a more problematic concept, insofar as it is may not be straightforwardly defined in behavioral terms. Specifically, it suggests that assumptions of rejecting pure rationality and self-interest from a behavioral economics perspective may be consistent with a selectionist perspective grounded in BA (i.e., selection of consequences). At the group level, theories and models of behavior in cultures and organizations may be expanded by a metacontingency analysis, which is able to capture the interdependency of behavior from a cultural selectionist perspective (e.g., Malott and Glenn, 2006; Carvalho Couto and Sandaker, 2016; Glenn et al., 2016; Wilson and Hayes, 2018).

## Conclusions

In summary, RFT offers an operational analysis of: (1) insensitivity to contingencies (i.e., resistance to change); (2) biases, insofar as maintaining coherence in the verbal networks; (3) measures of implicit cognition; and (4) frames of epistemological roots and manipulation of independent variables.

Nudging applies the principles of BE to route people’s choice behavior away from distortions attributable to the systematic effects of biases. According to RFT, biases are arbitrary relational responding behaviors, and “normal” products given a learning history that endures in a specific context. Similar to BE interventions, contextual manipulations aim to change human behavior either by intervening directly on the contingencies (contingency-shaped behavior) or by altering the relevant verbal variables (verbally governed behavior). Every context influences our choices, whether we are aware of it or not. This knowledge is common to BA, CBS, and BE.

BA and nudging share a common scope for addressing the effect of natural and social contingencies on situated choice behavior. We are only at the beginning, but the way ahead looks promising. As Furrebøe and Sandaker (2017) put it:

[T]he conceptual framework of behavior analysis enables investigation of the selection of functional relations between human choice behavior and the environmental contingencies of which it is a part. […] Behavioral economics provide good descriptions of important phenomena, and behavior analysis offers the technology to influence them. (p. 325)

Finally, RFT extends the vision of BA, by embedding it into CBS (Hayes et al., 2012). On a parsimonious level, RFT offers a functional-contextual theory that is capable to account for the wide variety of human overt and covert behaviors. It does so by using a restricted number of lower level interrelated principles, namely, operant classes of contextually controlled relational responding (Hayes et al., 2001). Thus, RFT rejects the postulation of internal homunculus-like constructs, submitting that all phenomena are accounted for because of context interdependency and exploitable variables. Truth criteria are based on how successful this manipulation is (Hayes et al., 2012); consequently, a contextual account of human behavior possesses not only an epistemological but also an immediate applicative value.

## Author Contributions

MT and VS wrote the first draft of the manuscript. MT, VS, and GP wrote sections of the manuscript. All authors contributed equally to reading, revising, and approving the submitted version of the manuscript.

### Conflict of Interest

The authors declare that the research was conducted in the absence of any commercial or financial relationships that could be construed as a potential conflict of interest.

## Footnotes

^1^For a thorough discussion of the properties that ethical nudging should possess, see also Sunstein (2015, 2016, 2018).
